# Surgical Outcomes after Arthroscopic Single Row Anchor Repair for Subscapularis Tears Concomitant with Injury of the Long Head of the Biceps Tendon

**DOI:** 10.1111/os.12649

**Published:** 2020-03-11

**Authors:** Youliang Shen, Xia Zhao, Chao Qi, Feng Chen, Haitao Fu, Yi Zhang, Yingze Zhang, Tengbo Yu

**Affiliations:** ^1^ Orthopaedic Center The Affiliated Hospital of Qingdao University Qingdao China; ^2^ Department of Orthopaedic Surgery Third Hospital of Hebei Medical University Shijiazhuang China

**Keywords:** Arthroscopy, Long head of the biceps tendon, Shoulder Joint, Subscapularis

## Abstract

**Objective:**

To analyze the clinical effects of single row anchor in repairing tears of the subscapularis muscle combined with the long head of the biceps tendon (LHBT) injury under arthroscopy.

**Methods:**

From June 2016 to June 2017, the clinical data of 32 patients with subscapularis combined with biceps tendon injury were retrospectively analyzed. Preoperative MRI examination of the shoulder joint was performed to evaluate tendon injuries, and the subscapularis muscle was repaired with single row anchor under arthroscopy, and tenotomy or tenodesis was performed on the long head tendon of the biceps humerus in the intertubercular groove. The range of motion and the functional score of the shoulder joint before and after the operation were evaluated. All patients were followed up for at least 24 months.

**Results:**

The mean follow‐up period was 28.8 months (range, 24–34 months). No infections occurred during the follow‐up period. The patients’ follow up exams showed significant improvement in postoperative shoulder joint flexion, external rotation, and internal rotation (*P* < 0.01), and the postoperative shoulder function American Society of Shoulder and Elbow Surgery Shoulder Joint Score (ASES; 80.6 ± 7.6) was significantly higher than the preoperative score (*P* < 0.01). The visual analog scale score (1.8 ± 0.8) was significantly lower than that before surgery (*P* < 0.01). The constant score (80.5 ± 7.4) was significantly higher than preoperation (*P* < 0.01). There was no significant difference in shoulder joint score between the tenotomy and tenodesis groups (*P* > 0.05). Preoperative and postoperative scores were, respectively: visual analog scale score (1.7 ± 0.9 vs 1.8 ± 0.0.8) ASES score (81.3 ± 7.9 *vs* 80.1 ± 8.0) and constant score (80.9 ± 8.0 *vs* 80.1 ± 6.9).

**Conclusion:**

Using single row anchor under arthroscopy to repair subscapularis combined with long head of biceps tendon injury yielded good results and high tendon healing rates were obtained.

## Introduction

The close relationship between the subscapularis tendon and the long head of the biceps tendon (LHBT) is known as the “brother structure.” They both play important roles in the maintenance of the dynamic stability of the shoulder joint^1^. In the past, most clinical studies reported that the incidence of subscapularis tears is very low. However, in many patients, subscapularis tears are missed. With the popularity of MRI and the development of arthroscopy, the diagnosis rate of subscapularis tears has increased^2^, ^3^. Subscapularis tears concomitant with the LHBT injury are the main cause of shoulder joint internal rotation pain. There has been an increase in arthroscopic repair of rotator cuff tears and Narasimhan *et al*. (2016) report the incidence of subscapularis tears under arthroscopic examination as 31.4%^4^. The subscapularis provides good stability for the LHBT, so subscapularis tears are often concomitant with LHBT lesions, with an incidence of approximately 63%^5^. Only open surgery was used for subscapularis repairs in the past. Retraction of the subscapularis tendon and the narrowing of the coracoid‐humerus space increase the difficulty of arthroscopic subscapularis repairs. However, with the development of new instruments and surgical techniques, the clinical outcomes of subscapularis repair under arthroscopy are significantly better than those of open surgery, and the possible additional injury of the LHBT can be treated at the same time. Only a few case reports or clinical studies (which have limited cohort sizes) on repair of subscapularis with LHBT exist in the literature.

The present study reviewed the retrospective data collected on Chinese patients who underwent arthroscopic subscapularis repair concomitant with LHBT tenotomy or tenodesis. The main purpose of this study was: (i) to determine the clinical and radiological outcomes of subscapularis tears concomitant with injury of the LHBT after surgery; and (ii) to report the surgical techniques of single row anchor repair for subscapularis tears concomitant with injury of LHBT. We hypothesized that: (i) the objective and subjective clinical outcome would be significantly improved at the final follow up; (ii) single row anchor repair for subscapularis tears concomitant with injury of LHBT would provide a better clinical and functional outcome; and (iii) there is a close relationship between the subscapularis tears and injury of LHBT.

## Materials and Methods

### *Patient Selection*

This study is retrospective, and the Ethics Review Committee of the Affiliated Hospital of Qingdao University approved the study.

#### *Inclusion Criteria*

The patients were eligible for inclusion in this study if they: (i) had subscapularis tears combined with LHBT injury; (ii) underwent shoulder arthroscopic surgery in our institution from June 2016 to June 2017; (iii) were followed for a minimum of 2 years; and (iv) were retrospectively recruited.

#### *Exclusion Criteria*

Exclusion criteria included: (i) massive rotator cuff tears with shoulder osteoarthritis; (ii) shoulder dislocation; (iii) proximal humeral fractures; and (iv) rheumatoid arthritis.

**Figure 1:**
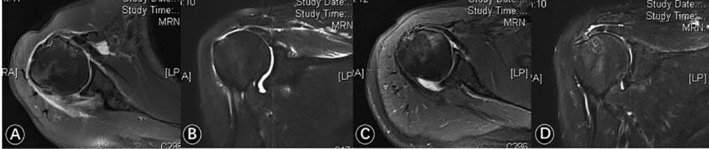
(A) Preoperative MRI showing subscapularis tears and long head of the biceps (LHB) tendon injury; (B) concomitant supraspinatus tears; (C) 6 months postoperatively, MRI showed that the subscapularis tendon healed well; and (D) MRI showed that the supraspinatus tendon healed well 6 months after surgery.

**Figure 2:**
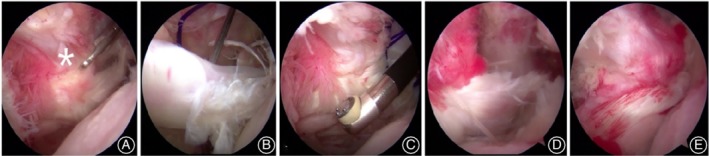
(A) Subscapularis tears, retraction, and comma sign (white star); (B) injury and dislocation of the long head of the biceps tendon; (C) one “PDS‐2” suture was used to draw the subscapularis and release the subscapularis arthroscopically; (D) sutures were passed through the subscapularis tendon after anchor implantation; and (E) the subscapularis after repair and the good coverage of footprint.

Given the criteria above, 46 patients with subscapular tears underwent arthroscopic surgery in our hospital. A total of 32 patients (19 men and 13 women) were confirmed by arthroscopy with injury of the LHBT. All patients underwent a preoperative X‐ray examination to exclude osteoarthritis and humeral head superior translation. MRI was used to confirm subscapularis tears and rotator cuff tears before surgery (Fig. 1A,B). The status of the subscapularis and LHTB was evaluated specifically using intraoperative arthroscopy (Fig. 2A,B).

### *Surgical Technique*

All operations were performed by the same surgeon. The surgery was performed under general anesthesia. The patient was placed in the lateral decubitus position, with the affected shoulder joint at 40° abduction and 20° forward flexion. The SPIDER shoulder positioner (Smith & Nephew) was applied for continuous traction. The posterior portal (2 cm below and 1–2 cm medial to the posterolateral corner of acromion) was routinely established as the viewing portal, and the anterolateral and anterior portals were successively established via the outside‐in technique. Exploration of the shoulder joint cavity, routinely using diagnostic arthroscopy, was performed to determine if the biceps tendon was also torn. In cases of subscapularis tendon retraction, it is necessary to correctly identify the “comma sign” (Fig. 2A). Then the camera was introduced into the subcoracoid space. Coracoid impingement was determined according to the preoperative measurements and if found during the operation, coracoplasty was performed, if necessary. The subscapularis was released adequately until the tendon was able to cover the entire footprint on the lesser tuberosity (Fig. 2C). After that, we refreshed the bone bed of the subscapularis footprint, and placed 1–2 absorbable suture anchors according to the extent of subscapularis tears. All limbs of the sutures were passed through the tendon using horizontal mattress sutures. Each suture used a transosseous equivalent double‐row configuration with polyether ether ketone (PEEK) swivel‐lock anchors (Fig. 2D,E).

**Figure 3:**
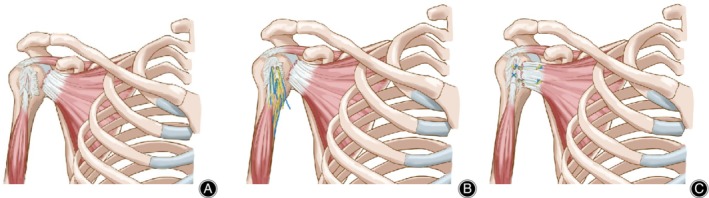
Schematic diagram of operating procedure: (A) subscapularis tears concomitant with injury of the long head of the biceps tendon (LHBT). (B) The position of the absorbable suture anchors of the subscapularis and the LHBT. (C) Sutures were passed through the subscapularis tendon after anchor implantation using horizontal mattress sutures. The LHBT was fixed at the intertubercular groove with one anchor by “lasso‐loop” suture.

The maximum extent of the extra‐articular LHB tendon was pulled into view using an arthroscopic probe, followed by evaluation of the biceps pulley and the superior labrum (Fig. 2B). For patients under 60 years old or requiring biceps tenodesis, the LHBT should be fixed at the intertubercular groove with one anchor by “lasso‐loop” suture. For patients older than 60 years, the tenotomy of LHBT was performed at the proximal origin of the posterosuperior labrum at the supraglenoid tubercle with an arthroscopic punch. In this study, LHBT tenotomy (20 cases) and biceps tendon tenodesis (12 cases) were performed. In patients with massive rotator cuff tears, the LHBT can be used as a “patch” to cover the bare area of the footprint after partial rotator cuff repairs (Fig. 3).

### *Postoperative Rehabilitation*

After surgery, the affected limb was suspended for 6 weeks at adduction and internal rotation position, external rotation >0°, and overtop movement was prohibited for 6 weeks; only passive elbow movement was allowed. After 6 weeks, passive shoulder external rotation and overtop arm lifting were permitted. Muscle strength exercises were started at 12 weeks, and higher intensity muscle strength exercises were started at 24 weeks.

### *Evaluation Criteria*

All patients were examined for range of motion of shoulder joints before surgery and at the last follow up. This included flexion forward, external rotation and internal rotation. The incidence of the Popeye sign was recorded. The visual analog scale (VAS) score, the American Society of Shoulder and Elbow Surgery Shoulder Joint Score (ASES), and the Constant score were used to evaluate the function and postoperative pain before surgery and at the last follow up. Six months after surgery, the patients underwent MRI examination to evaluate tendon healing and re‐tear rates (Fig. 1C,D).

#### *Visual Analog Scale for Pain*

The VAS is the most commonly used questionnaire for quantification of pain. It is a continuous scale comprised of a horizontal or vertical line, usually 10 cm in length. For pain intensity, the scale is most commonly anchored by “no pain” (score of 0) and “pain as bad as it could be”(score of 10). A score of 0 is considered as no pain, 1–3 mild pain, 4–6 moderate pain, and 7–10 severe pain^6^.

#### *Constant Score Scale*

The Constant score is by far the most commonly used method for evaluation of rotator cuff tears. The 100‐point scoring scale takes into account both subjective and objective measurements: pain (0–15, with 0 being maximal pain and 15 no pain); activities of daily living (4 × (0–5) = 0–20, 0 worst and 5 best for each item); mobility (4 × (0–10) = 0–40, active, pain‐free range of elevation: +2 points per 30°, where 0 is worst and 10 is best for each item; position of hand: 0 worst to 10 best); and strength (0–25, 1 point per 0.5 kg, maximum 25 points). A total score of 0 is worst and 100 is best function.^7^

#### *American Society of Shoulder and Elbow Surgery Shoulder Joint Score*

The ASES score was developed by the Society of the Shoulder and Elbow Surgeons, including a patient self‐assessment section (patient ASES [pASES]) and a section completed by the examiner (clinical ASES [cASES]). The cASES section includes a physical examination and documentation of range of motion, strength and instability, and demonstration of specific physical signs. No score is derived for this section. The pASES has 11 items that can be used to generate a score. These are divided into two areas: pain (1 item) and function (10 items). The severity of pain is scored by VAS. The 10 items in the function area include activities.^8^

### *Statistical Analysis*

All statistical analyses were performed using SPSS software for Windows version 22 (SPSS, Chicago, IL, USA). A paired *t*‐test was performed to assess the difference in shoulder function score before and after surgery. A *P*‐value of <0.05 was considered to represent a significant difference.

## Results

### *Follow‐up*

This case series included 32 patients, 13 women and 19 men, and the mean age was 53 ± 11 years (range 32–69). All 32 patients were postoperatively followed up for 24–34 months, with an average of 28.8 months.

### *General Results*

The mean operative time was 110.45 ± 30.23 min. Arthroscopic Lafosse classification of subscapularis tears^9^, LHTB injury, and the degree of rotator cuff tears are shown in Table 1.

**Table 1:** Arthroscopic subscapularis tear classification and concomitant injuries

	percentage (%)
Lafosse classification of subscapularis tears	
II	9 cases (28.1%)
III	17 cases (53.1%)
IV	6 cases (18.7%)
Concomitant rotator cuff tears	
None	4 cases (12.5%)
Supraspinatus tears	23 cases (71.8%)
Supraspinatus and infraspinatus tears	5 cases (15.6%)
The long head of the biceps tendon injury	
Inflammation	8 cases (25.0%)
Injury	19 cases (59.3%)
Dislocation or subluxation	5 cases (15.6%)

### *Clinical Improvement*

#### *Shoulder Joint Range of Motion*

**Figure 4:**
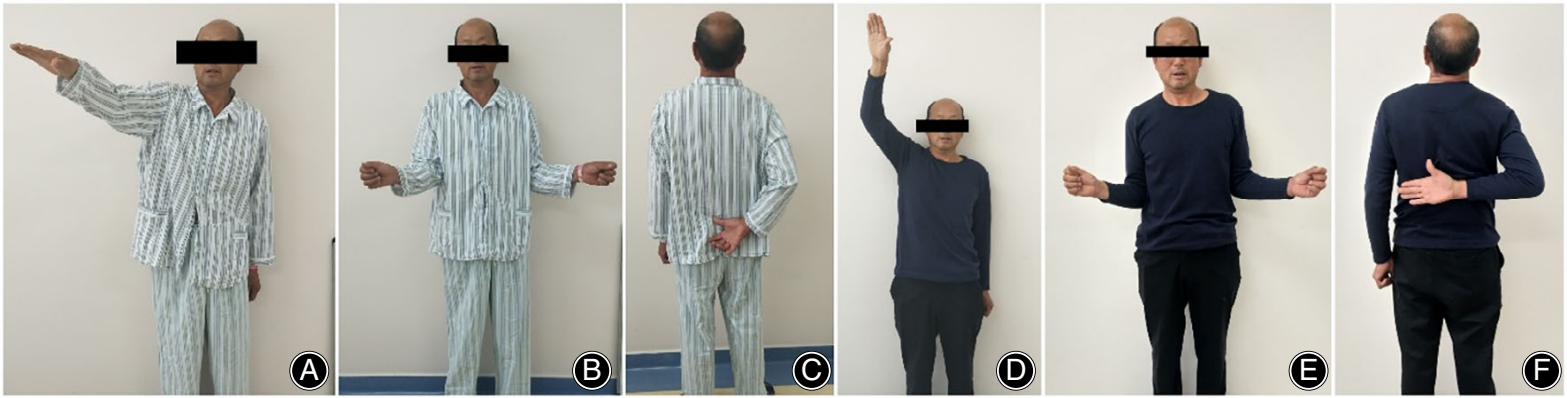
A case of subscapularis tears concomitant with injury of the long head of the biceps tendon. A 52‐year‐old male farmer, with traumatic injury: (A–C) physical examination before operation; and (D–F) physical examination at the final follow‐up.

Before the surgical intervention, the mean flexion forward, the external rotation, and the internal rotation of the shoulder were 109.8° ± 17.2°, 58.1 ± 15.8, and L3 (S1‐T12), respectively (Fig. 4A–C). At the last follow up after the operation, the mean flexion forward, the external rotation, and the internal rotation were significantly improved to 146.7° ± 9.0° (*t* = 10.75), 70.5 ± 12.6 (*t* = 19.34), and T11 (L1‐T8), respectively (Fig. 4D–F; *P* < 0.001, Table 2). The function of shoulder joint ROM and shoulder function score was significantly improved after surgery in different types of subscapularis tears (*P* < 0.005, Table 3).

**Table 2:** Comparison of shoulder function scores

	Re‐operation	Last follow‐up	*t*‐value	*P*‐value
Shoulder joint ROM				
Flexion forward (°)	109.8 ± 17.2	146.7 ± 9.0	10.75	<0.001
External rotation (°)	58.1 ± 15.8	70.5 ± 12.6	19.34	<0.001
Internal rotation	L3 (S1‐T12)	T11 (L1‐T8)		
Shoulder function score				
VAS score	6.2 ± 1.1	1.8 ± 0.8	18.52	<0.001
ASES score	46.3 ± 6.4	80.6 ± 7.6	19.50	<0.001
Constant score	46.0 ± 5.7	80.5 ± 7.4	20.87	<0.001

ASES, American Society of Shoulder and Elbow Surgery Shoulder Joint Score; ROM, range of motion; VAS, visual analog scale.

**Table 3:** Comparison of shoulder joint function scores in different type of subscapularis tear subgroups

Type of tear		Shoulder Joint ROM			Shoulder Function score			
		Re‐operation	Last follow‐up	*P*‐value		Re‐operation	Last follow‐up	*P*‐value
II	Flexion forward (°)	117.9 ± 19.4	150.9 ± 8.2	<0.05	VAS	5.9 ± 1.3	2.0 ± 0.9	<0.05
	External rotation (°)	52.1 ± 6.0	74.0 ± 5.3	<0.05	ASES	47.2 ± 8.7	86.0 ± 6.7	<0.05
	Internal rotation	L2 (S1‐T12)	T10 (L1‐T8)		Constant	45.0 ± 6.7	84.6 ± 5.8	<0.05
III	Flexion forward (°)	104.8 ± 16.8	145.0 ± 9.84	<0.05	VAS	6.3 ± 1.0	1.8 ± 0.7	<0.05
	External rotation (°)	51.0 ± 5.7	77.8 ± 4.2	<0.05	ASES	45.2 ± 6.0	77.3 ± 7.0	<0.05
	Internal rotation	L4 (S1‐L2)	T11 (L2‐T9)		Constant	46.5 ± 5.4	77.8 ± 7.2	<0.05
IV	Flexion forward (°)	111.5 ± 11.3	145 ± 6.4	<0.05	VAS	6.5 ± 1.4	1.33 ± 0.5	<0.05
	External rotation (°)	56.5 ± 2.3	79 ± 1.8	<0.05	ASES	46.0 ± 5.8	82.0 ± 6.5	<0.05
	Internal rotation	L5 (S1‐L3)	L2 (L4‐T12)		Constant	45.6 ± 5.4	81.7 ± 7.7	<0.05

ASES, American Society of Shoulder and Elbow Surgery Shoulder Joint Score; ROM, range of motion; VAS, visual analog scale.

#### *Functional Evaluation*

##### Visual Analog Scale for Pain

The VAS score was remarkably decreased, from preoperative 6.2 ± 1.1 to postoperative 1.8 ± 0.8 points at the final follow‐up, indicating that the pain was significantly reduced postoperatively (*t* = 18.52, *P* < 0.001, Table 2).

##### Constant Score

Clinical assessment showed that the preoperative and postoperative Constant scores were 46.0 ± 5.7 and 80.5 ± 7.4 points, respectively, and significant difference existed between them (*t* = 20.87, *P* < 0.001,Table 2).

##### American Society of Shoulder and Elbow Surgery Shoulder Joint Score

Before the surgical intervention, the mean ASES score of the patients was 46.3 ± 6.4 points. At the last follow up after the operation, the mean ASES score was significantly improved, to 80.6 ± 7.6 (*t* = 19.50, *P* < 0.001,Table 2).

### *Radiographic Outcomes*

MRI examination confirmed that subscapularis tendons had healed well 6 months after surgery in 29 patients and re‐tears occurred in 3 patients, with an incidence of 9.6%. The data for the patients with re‐tears are shown in Table 4.

**Table 4:** The data of 3 patients with subscapularis re‐tear

	Case 1	Case 2	Case 3
Gender	Man	Woman	Man
Age (year)	56	67	65
Occupation	Manual labor	Retired	Manual labor
History of trauma	Yes	No	Yes
Course (month)	13	26	21
Lafosse classification	III	IV	IV
Concomitant rotator cuff tears	IST + SST	SST	SST
Treatment of LHBT	Tenodesis	Tenotomy	Tenotomy

IST, infraspinatus tendon; LHBT, long head of the biceps tendon; SST, supraspinatus tendon.

### *Complications*

All incisions healed well, and no infections, nerve injuries, or fractures occurred. The Popeye deformity occurred in 5 patients in the tenotomy group, but there was no pain and there was no significant decrease in shoulder joint function in the 5 patients (Table 5).

**Table 5:** Comparison of shoulder joint function scores in tenotomy and tenodesis

	Tenotomy (20 cases)	Tenodesis (12 cases)	*P*‐value
Gender (female/male)	8/12	5/7	—
Age (year)	58.7 ± 9.3	43 ± 6.2	—
BMI (kg/m^2^)	29.11 ± 3.3	27.44 ± 4.2	—
Operated side (L/R)	13/7	7/5	—
VAS score	1.58 ± 0.19	1.90 ± 0.17	*P* = 0.254
ASES score	81.75 ± 2.25	79.9 ± 1.68	*P* = 0.523
Constant score	81.50 ± 2.41	79.85 ± 1.54	*P* = 0.549

ASES, American Society of Shoulder and Elbow Surgery Shoulder Joint Score; BMI, body mass index; VAS, visual analog scale.

## Discussion

Arthroscopic subscapularis repair has been reported to obtain good results in terms of relieving pain and improving shoulder joint function, as well as in treating concomitant shoulder lesions, such as rotator cuff tears^10^. From June 2016 to June 2017, 46 cases of subscapularis tears had undergone arthroscopic surgeries at our hospital, of which 32 cases, during arthroscopy, were diagnosed concomitant with LHBT injury, with an incidence rate of up to 69.5%. In this study, the 32 patients with subscapularis tears and LHBT injury, addressed by arthroscopic single row anchor repair, had significant improvement in shoulder joint function. We found that arthroscopic repairs of subscapularis tears yielded favorable and stable clinical outcomes, with minimal invasiveness and cosmetic enhancements.

However, this procedure for subscapularis repair is relatively difficult and requires experienced surgical skills due to the narrowed subcoracoid space. Attention should be paid to the following during this procedure:

(i) The difficulty of identifying the subscapularis tendon under arthroscopy in the cases of chronic subscapularis tears due to retraction of the tendons. Therefore, clear identification of the “comma sign”^11^ formed by SGHL and CHL torn from humerus less tuberosity must be noted. Once identified, one “PDS II” suture should pass through the “comma” structure as a traction suture, and then the freeing of the middle glenohumeral ligament and the adhesive tissues surrounding subscapularis must be carefully performed under traction with radiofrequency. At least one limb of the anchor sutures should be passed through the “comma” structure to decrease the tension of subscapularis after fixation and to reduce the re‐tear rate.

(ii) Coracoid, as the “lighthouse” structure in the front of the shoulder: there are many important vascular and neural structures medial to the coracoid process. The release of the subscapularis tendon ought to be performed laterally and posteriorly to the coracoid, with the operation carried out under direct view.

(iii) If the retraction stress of the subscapularis tendon is extremely high, the humeral attachment of the subscapularis could be moved to the medial side. In Patrick and Stephen (2012), 4–7 mm of medial migration of the subscapularis footprint did not affect the postoperative shoulder joint function^12^.

(iv) Subcoracoid decompression should be used, if necessary. Osti reported that the narrowed subcoracoid space caused by the abnormal structure and the hyperplasia of the coracoid is the main reason for the subscapularis tears^13^. Friedman evaluated the distance between the coracoid process and the humerus with MRI and demonstrated that the average distance of patients without subcoracoid impingement symptoms was 11 mm, while the average distance was less than 5.5 mm in patients with impingement symptoms; this is significantly less than that of the normal group^14^. Because it is difficult to accurately measure the distance between the coracoid process and the subscapularis tendon when the subscapularis tendon is completely torn, our experience is to perform the subcoracoid decompression when the distance between the coracoid process and the humerus is less than 6 mm preoperatively.

(v) If the subscapularis tear is larger than two‐thirds or is completely torn, two anchors should be used for repair; if the tear is less than one‐third, one anchor can be used for repair.

Among the 32 patients in this study, subscapularis re‐tear was detected in 3 cases, accounting for 9.6%, of which 2 cases involved heavy workers. Jo *et al*. reported that the anatomical characteristics and the type of subscapular tears were related to postoperative re‐tears^15^. The upper two‐thirds of the subscapularis is attached to the lesser tuberosity as a form of tendon bone, while the lower one‐third is attached directly to the lower side of the lesser tuberosity as a form of muscle bone. The lower one‐third of the subscapularis muscle plays important roles in preventing pseudoparalysis and in maintaining stability of the glenohumerus. Therefore, the muscle part of the lower third of the subscapularis contributes to reducing the incidence of re‐tears^16^. Some have reported that the subscapularis re‐tear rate is approximately 10% regardless of the extent of the tears and the technique used for repairs^17^. Yoon demonstrated that both arthroscopic single row repair and double row suture bridge repair of separated full‐thickness subscapularis tears achieves satisfactory clinical effect and a high tendon healing rate, but patients with significant fat infiltration of the subscapularis muscle were excluded in their study^18^. Fandridis showed that the high degree of subscapularis atrophy and severe fat infiltration before surgery were negative factors in the impact on clinical prognosis^19^. In this study, there were 3 cases of subscapularis re‐tears, of which 2 cases had subscapularis muscle atrophy and obvious fat infiltration before surgery, and 2 cases involved heavy workers. Heavy physical labor or competitive sports may also play a role in re‐tears^15^. Therefore, both patients’ compliance in and proper rehabilitation exercises after repair of subscapularis tears are particularly important.

Because of the special anatomical relationship between the subscapularis and the LHTB, the subscapularis tears often lead to injury of the medial suspension structure of the biceps tendon and subsequently cause instability of the biceps tendon. Therefore, subscapularis tears should be highly suspected if LHBT dislocation is found during surgery. If management of the LHBT injury is ignored, the postoperative pain will not be relieved and the surgical outcomes will be influenced. Currently, there is controversy as to which is better, LHBT tenotomy or tenodesis. LHBT tenotomy has the advantages of less complex surgery, being economical, fast recovery times, and fewer complications. However, some other people think that the Popeye sign is more likely to appear after tenotomy. This will affect aesthetics, so tenodesis should be performed, and the tenodesis should match the biomechanical properties of shoulder and elbow joints better^20^. Moreover, rotator cuff repair with LHBT augmentation has been suggested as an alternative repair technique for large to massive RCT that cannot be treated with complete repair^21^, ^22^. This technique enables wider footprint coverage, with the augmented LHBT acting as an internal splint. Cho found that patients in the LHBT augmentation group demonstrated a significantly better healing rate on MRI at a mean follow up of 15 months when compared with the partial repair group (58% *vs* 26%, *P* < 0.036)^23^. In this group of 32 patients, 20 patients underwent tenotomy (5 patients developed the Popeye sign) and 12 patients underwent tenodesis (12 patients with no Popeye sign). There was no significant difference in postoperative pain score and pain duration. However, the incidence of the Popeye sign was significantly increased after tenotomy. Therefore, patients preparing to undergo tenotomy of LHBT should be informed of the possibility of the Popeye sign preoperatively.

### *Limitations of the Study*

One limitation to this study is that the number of patients was relatively small. Moreover, retrospective data collection and analysis were conducted, which could have allowed for patient selection bias.

### *Conclusion*

In conclusion, attention should be paid to avoid missed diagnoses of subscapularis tears and injuries of LHBT under arthroscopy. The use of arthroscopic single row repairs for subscapularis tears combined with LHBT injury achieved good surgical results and a lower rate of re‐tear. In regard to tenotomy and tenodesis of LHBT, the clinical outcomes have no significant difference, so the decision on which to use needs to be based on the patient's age, patient requirements, and other factors.
